# “Effectiveness of interventions in reducing pain and maintaining physical activity in children and adolescents with calcaneal apophysitis (Sever’s disease): a systematic review”

**DOI:** 10.1186/1757-1146-6-16

**Published:** 2013-05-03

**Authors:** Alicia M James, Cylie M Williams, Terry P Haines

**Affiliations:** 1 Podiatry, Peninsula Health Service, Melbourne, Australia; 2 Allied Health Research Unit, Southern Health, Melbourne, Australia; 3 Monash University, Melbourne, Australia

## Abstract

**Background:**

Calcaneal apophysitis, also commonly known as sever’s disease, is a condition seen in children usually aged between 8–15 years. Conservative therapies, such as taping, heel lifts and orthotic intervention are accepted management practices for calcaneal apophysitis, though there is very little high quality research examining the efficacy of such treatment modalities. Previous narrative literature reviews and opinion pieces provide some evidence for the use of heel raises or orthoses. The aim of this manuscript was to complete a systemic review on the treatment options for calcaneal apophysitis as measured by pain reduction and maintenance of physical activity.

**Methods:**

A search strategy completed by two reviewers examined nine databases from inception to May 2012. Search terms included heel pain, children, adolescent, calcaneal apophysitis, sever’s disease, treatment, and management (full text publications, human studies). Systematic reviews, randomised control trials, case series, and case studies were included. The reference lists of the selected articles were also examined. The methodology, quality and risk of bias was examined and assessed using the PEDro scale.

**Results:**

Nine articles were retrieved including three clinical trials involving randomisation, two case series, two retrospective case reviews, and two case reports. Effect size calculations and a meta analysis were unable to be completed due to the limited data reported within the literature. Numerous treatment options were reported throughout the literature, though few were examined against a control or alternate treatment option in well-designed trials. The limited evidence indicated that orthoses provided greater short-term pain relief than heel raises. Health practitioners should view these results with caution, as there were apparent methodological problems with the employed study design and limited follow-up of participants.

**Conclusion:**

There is limited evidence to support the use of heel raises and orthoses for children who have heel pain related to calcaneal apophysitis. Further research is needed to generate higher quality evidence with larger sample sizes, and validated measures of pain and function to establish effective treatment approaches for children with calcaneal apophysitis.

## Background

Early in the 20th century, Sever reported a condition characterised by pain in the posterior and inferior region of the heel in very active and/or overweight children. This was reported to be characterised by the enlargement of the epiphyseal line of the ossific nucleus of the calcaneus on radiographic examination, with cloudiness and obliteration of the epiphyseal line [1]. Over a decade later, an explanation for the cause of calcaneal apophysitis was posited by Lewin [2], who argued that it was inflammation caused by traction in opposite directions between the achilles tendon and the plantar fascia and aponeurosis.

Posterior heel pain classified as calcaneal apophysitis or sever’s disease may be a common musculoskeletal injury in children as this condition has been reported to account for between 2% - 16% of presentation at sports clinics [3-5]. Calcaneal apophysitis is reported as a self limiting condition [6], usually presenting between the ages of 8–15 years [7,8], but has been observed in children as young as six [9]. The pain related to this inflammation is though to cease after fusion of the calcaneus [1]. However, no studies have yet reported the incidence or prevalence of this condition in the general population [10]. Pain with walking and sport is often reported in this condition and is a cause of concern for both parent and child. The physical activity reported to produce the highest levels of pain include frequent running and jumping such as soccer [11]. In rare cases, it has been reported that untreated calcaneal apophysitis can cause calcaneal apophyseal avulsion fractures [12]. Beyond the pain and physical disability associated with calcaneal apophysitis, children with this condition have also been found to have lower ‘Happiness’ and lower ‘Sport/Physical Function’ subscale scores from the Paediatric Orthopaedic Surgeons of North America ‘Musculoskeletal Quality of Life’ questionnaire (n = 67), when compared to children without calcaneal apophysitis (control group n = 236) [13].

The recommended treatment options for calcaneal apophysitis are varied. Only one literature review has examined literature pertaining to calcaneal apophysitis between 2008–2011, this review finding that there is no criterion based treatment path and recommended further evaluation of treatment methods [14]. Narrative literature reviews [15,16] have recommended the following treatment options: rest or cessation of sport [17-23], heel lift, orthoses, mobilisation [1,17,19,21,23-27] stretching or strengthening exercise programs [1,2,17,19-21,23,24,28], padding for shock absorption or taping of heel [1,2,19,27-31], ultrasound/pharmaceutical prescriptions/ice [3,11,19-21], immobilisation casting and crutches [11,25,26,32] and footwear prescription with appropriate support and cushioning [33]. This review aims to synthesise the available evidence on the efficacy of treatment approaches for maintaining physical activity and reducing pain (short and long term) for children with calcaneal apophysitis.

## Method

### Development of a clinical question

The clinical question for this systematic review was generated using the PICO format [34]. It was: ‘In patients with calcaneal apophysitis (sever’s disease) is there an effective treatment that relieves pain and maintains physical activity?

This question was separated into search terms and nine electronic databases were searched (Medline, CINAHL, Pubmed, Web of Science, Scopus, Ebscohost, Google scholar, Physiotherapy Evidence Database (PEDro) and the Cochrane Library) from the earliest available date until May 2012 using key word search terms (Table 1). Given the small amount of literature on this condition, terms for the disease and generic treatment were searched without limiting the search using specific treatment or outcome terms.

**Table 1:** Search strategy results

**Search terms**	**Medline**	**CINAHL**	**Web of Science**	**Pub Med**	**Scopus**	**Ebsco host**	**PEDRO**	**Cochrane library**
Heel pain and children	23	18	111	189	284	69	0	4
Heel pain and Adolescent	3	6	22	195	288	108	0	0
Calcaneal apoph*	37	10	29	26	39	39	0	0
Calc* apoph*	22	9	34	32	54	49	3	0
Sever’s disease	31	23	25	26	37	65	0	0
Calcaneal apoph* and treatment	7	3	14	19	21	4	0	0
Sever’s disease and Treat*	7	7	8	14	16	14	0	0
Calcaneal apoph* and manage*	6	0	7	6	4	2	0	0

* truncation character that allows the retrieval of varying endings of your search term.

### Search strategy results

Two authors (AJ and CW) independently reviewed all the retrieved studies against the eligibility criteria (Table 2). Full articles were obtained where there was uncertainty from the abstract. The reference list of each of the articles was also reviewed and any articles meeting the inclusion criteria were also included.

**Table 2:** Inclusion/exclusion criteria

**Inclusion criteria**	**Exclusion criteria**
*Design:*	• Articles not published in English
• RCT	• Articles not including treatment
• Clinical trial	• Non peer reviewed publications
• Case report,	• Author opinion
• Case series	
*Participants:*	
• Children aged 6–15 years	
• Diagnosis of CA/Sever’s disease	
*Intervention:*	
• Orthoses	
• Heel lifts	
• Stretching	
• Icing	
• Strapping	
• Other treatment modalities	
*Outcome measure*:	
• Pain	
• Physical activity (sporting activities)	

**Figure 1:**
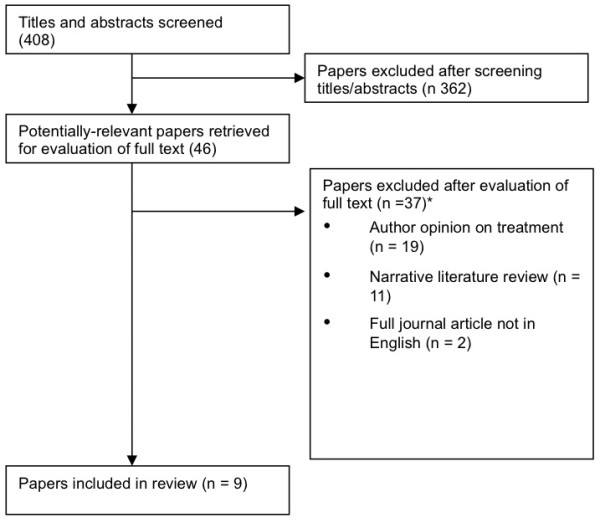
Review of literature.

Four hundred and eight articles were extracted; from this three hundred and sixty two were excluded as per the inclusion/exclusion criteria. Following review of the full text, eleven texts were found to be significant, nine publications were included within the review (Figure 1). Two publications were excluded: a systematic review that was not published in a peer review journal which examined the literature between 2008–2011 [14], and a masters thesis examining treatment options which was written in Spanish [35].

### Data extraction

All articles included within the review underwent methodological assessment using the PEDro scale. This scale [36] was used by the two authors independently, who following this, met in person to discuss and resolve any disagreement. Articles relating to treatment of calcaneal apophysitis were also classified into levels of evidence using criteria set out by the Oxford Centre for Evidence Medicine [37]. This system recommends that the most appropriate research to guide treatment are systematic reviews of randomised control trials (Level 1), randomised control trials (Level 2), non-randomised controlled cohort/follow up study (Level 3), cohort studies and/or case series (Level 4) and mechanism based reasoning (Level 5) [37].

## Results

### Description of studies

A summary of the articles identified has been compiled within Table 3. Three articles describing clinical trials involving randomisation (Level 2) were found, of which two compared heel raises to orthoses in a cross over design of randomisation [38,39] while the other article described comparison of orthoses to no treatment (control) [40]. The same author group conducted all three studies. An article describing a cohort study (Level 3) was also found which reported on many concurrent treatment modalities [41]. The remaining four articles were case series studies (Level 4), which also used many concurrent treatment modalities [17,42-45]. The majority of studies were found to have low scores on the PEDro scale (Table 4).

**Table 3:** Summary of studies included

**First Author, Year**	**Study design**	**Country/Population**	Diagnosis *	**Sample size**	**Treatments used**							**Outcome measurement**	**Assessment time frame**	**Effect of intervention**	**Level of evidence**[37]
					**Rest**	**Ice**	**Stretch**	**Taping**	**Heel Lifts**	**Orthoses**	**Other**				
Hunt, 2007 [42]	Case series	USA	Yes	11	*X*	*X*	*X*	*✓*	*X*	*X*	*X*	11 point pain scale	5 minutes	P = .001	4
		9 male, 2													
		female													
		Aged 9-14													
Kvist, 1991 [41]	Retrospective case review	Finland	Yes	67	*✓*	*✓*	*✓*	*X*	*✓*	*✓*	*✓*	Pain^**^	16 weeks	Not reported	3
		36 Male									*(Massage)*	Activity history			
		31 female													
		Aged 8-16													
Leri, 2004 [43]	Case Report	USA	Yes	1	*✓*	*✓*	*✓*	*X*	*X*	*X*	*✓*	Pain^**^	1 week	Not reported	4
		1 Male													
		Age 11													
Micheli 1987 [17]	Retrospective case review	Sweden	Yes	85	*X*	*X*	*✓*	*X*	*✓*	*✓*	*✓*	Symptomatic relief	48 weeks	Not reported	3
		64 Male									*mobil/activity mod*	Time			
		21 Female										Activity history			
		7-15 years													
Perhamre 2011 [38]	Randomised Trial	Sweden	Yes	35	*X*	*X*	*X*	*X*	*✓*	*✓*	*X*	Borg CR-10 Pain	8 weeks	IQR	2
		35 males												P vaues	
		Aged 9–15 years													
Perhamre 2011b [40]	Randomised Trial	Sweden	Yes	30	*X*	*X*	*X*	*X*	*X*	*✓*	*X*	Borg CR-10-Pain	4 weeks	P values	2
		30, 45 or 50 children										Endgstrom Activity level			
		Aged 9–15 years													
															
Perhamre 2010c [39]	Randomised control trial	Sweden	Yes	51	*X*	*X*	*X*	*X*	*✓*	*✓*	*X*	Borg CR-10- Pain	26 weeks	P Values	2
		51 males													
		Aged 9-15													
White,	Case report	USA	Yes	1	*✓*	*✓*	*✓*	*X*	*✓*	*X*	*✓*	VAS Pain	18 days	Not reported	4
2006 [44]		1 female									*(NSAIDS, Heat, mobilised)*	LEFS^***^			
		Aged 8										Strength			
												ROM			
Wooten , 1990 [45]	Case Series	USA	Yes	5				X	*X*	*X*	*Tapping padding*	Pain ^**^	4 weeks	P Values	4
		5			*X*	*✓*	*✓*					ROM			

* Diagnosis of calcaneal apophysitis confirmed by medial and lateral compression (calcaneal squeeze test).

**The pain scale utilised was not disclosed.

***LEFS- Lower extremity functional screen.

**Table 4:** PEDro scores of included studies

**First Author and Year**	**Study design**	**Eligibility criteria specified.**	**Random allocation**	**Concealed allocation**	**Group similar at baseline**	**Participant blinding**	**Therapist blinding**	**Assessor blinding**	**<15% dropouts**	**Intention to treat analysis**	**Between group difference reported**	**Point estimate and variability reported**	**Total**
**Hunt, 2007**[42]	Case series	Yes	*X*	*X*	*✓*	*X*	*X*	*X*	*✓*	*✓*	*X*	*X*	**3/10**
**Kvist, 1991**[41]	Retrospective case review	yes	*X*	*X*	*X*	*X*	*X*	*X*	*X*	*N/A*	*X*	*X*	**0/10**
**Leri, 2004**[43]	Case Report	No	*X*	*X*	*X*	*X*	*X*	*X*	*X*	*✓*	*X*	*X*	**1/10**
**Micheli 1987**[17]	Retrospective case review	yes	*X*	*X*	*X*	*X*	*X*	*X*	*X*	*X*	*X*	*X*	**0/10**
**Perhamre 2011a**[38]	Randomised Trial	*Yes*	*✓*	X*	*X*	*X*	*X*	*X*	*✓*	*X*	*✓*	*✓*	**4/10**
**Perhamre 2011b**[39]	Randomised Trial	*Yes*	*✓*	X *	*✓*	X	*X*	*X*	*✓*	*X*	*✓*	*✓*	**5/10**
**Perhamre 2011c **[40]	Randomised trial	*Yes*	*✓*	*X*	*X*	*X*	*X*	*X*	*✓*	*X*	*✓*	*✓*	**4/10**
**White, 2006**[44]	Case report	No	*X*	*X*	*X*	*X*	*X*	*X*	*X*	*✓*	*X*	*X*	**1/10**
**Wooten , 1990**[45]	Case Series	Yes	*X*	*X*	*X*	*X*	*X*	*X*	*X*	*✓*	*X*	*✓*	**2/10**

♦ Concealment was reported to be tickets concealed within a box. Minimum concealment is recommended to be as sequential numbered, opaque sealed envelope to minimise biases and confounding variables [46].

Criterion 1- Subjects were randomly allocated to groups.

Criterion 2- Allocation was concealed.

Criterion 3- The groups were similar at baseline regarding the most important prognostic indicators.

Criterion 4- There was blinding of all subjects.

Criterion 5- There was blinding of all therapists who administered the therapy.

Criterion 6- There was blinding of all assessors who measured at least one key outcome.

Criterion 7- Measure of at least one key outcome were obtained from more than 85% of the subjects initially allocated to groups.

Criterion 8- All subjects for whom outcome measures were available received treatment or control condition as allocated or when this was not the case, data for at least one key outcome was analysed by ’intention to treat‘.

Criterion 9- The results of between group statistical comparisons are reported for at least one key outcome.

Criterion 10- The study provides both point measures and measures of variability for at least one key outcome.

### Meta analysis/pooling of data

A meta analysis of the selected nine articles was not completed as pooling was restricted by inadequate statistical analysis/reporting, missing data and dissimilar interventions. Three author groups [40,42,45] were contacted by email and requested to provide outcome measure data clarification in a format that would allow for meta analysis, however this was unable to be garnered.

The treatment recommendations from this review were grouped into two general categories for presentation of results:

i) Strategies aimed at minimising the inflammation process, minimising pain and promoting the healing process. These included, modified rest or cessation of sport [41,43], pharmaceuticals [44] and/or ice [41,43,44].

ii) Mechanical strategies aimed at modifying biomechanical factors that may contribute to calcaneal apophysitis. These included the use of heel raises [17,38,39,41,44], taping/padding [42,45], orthoses [17,38-41], and stretching of the gastrocnemius / soleus / achilles tendon complex [17,41,43,44].

### Minimising the inflammation process

The use of ice, stretching and rest or restriction of activities has been incorporated in the majority of studies [41,43,44]. These were commonly provided, as a cluster of treatments and the individual effectiveness of each modality have not been examined within the literature. Even when provided as a cluster of treatments, none of the studies identified in this review reported the results of the effectiveness of this treatment on pain or physical activity levels.

#### Non steroidal anti-inflammatory drugs (NSAIDS)

There was one study within the review that incorporated the use of NSAIDS for this condition. This article reported the effectiveness of a topical NSAIDS in the reduction of pain in a single case report . While the use of this particular topical NSAIDS, Ketroprofen, was contraindicated in children under the age of 12 [47] this isolated case was conducted with medical involvement and was well monitored for adverse effects. There were confounding factors reported throughout this study, as the single participant also had hot/cold therapy and mobilisation. It is not known how long the impact of this therapy lasted, nor is it known if the decrease in pain was achieved through the application of topical NSAID or from the physical therapy modalities.

### Modifying biomechanical factors

#### Taping

The use of taping alone [42] was only reported in one pre-post intervention, (n = 10) case series. This modality was reported by the authors to be effective in the acute and immediate (no time frame was reported) relief of pain with p = 001. The measurement of pain was by an 11-point ordinal scale with 0 representing ‘absolutely no pain’ and 10 representing ‘worst imaginable pain’. The wording of the pain question was not provided so it is unclear which domain within the construct of pain was being measured.

The use of padding and strapping was utilised [45] in one case series, (n = 11) where n = 5 had a diagnosis of calcaneal apophysitis. The authors reported this modality to be effective in decreasing pain during and post activity across a time period of 1 month with p = <0.01. As this study also included adults who had posterior heel pain, the resultant pain relief from this treatment should be cautiously regarded.

#### Orthoses

The use of orthotics has been reported within a number of studies and for the purposes of this review, all of the devices that were custom made, moulded around the heel, with or without an arch support are termed as orthoses. There were five publications identified in this review [17,38-41], which examined the efficacy of orthoses either in comparison to heel lifts or no intervention. Two of these described retrospective case note reviews, while three other publications described two randomised control trials.

In the retrospective case studies examining use of orthoses [17,41], the type or style of orthoses used was not described and specific data and/or statistical analysis that would permit evaluation of the efficacy of this intervention were not provided.

The remaining three papers reported the results of two randomised control trials, conducted by the same investigators. The first randomised trial [38,39] was a cross-over design with a sample size of 44. There was an initial two-week observation period with no intervention, followed by a four-week ‘intervention’ period where participants were randomly assigned to orthoses or heel raise interventions. This was followed by another two-week period with no intervention, and another four-week ‘intervention’ period with the opposite intervention compared to the first intervention period. Finally, there was another two-week period with no intervention. At the end of this trial, the investigators allowed participants to choose which intervention they preferred and then re-assessed the participants twelve weeks later. Outcomes in this research were measured at the end of each ‘no intervention’ and ‘intervention’ period. While there was no control group within this study, the cross-over design is considered to be the same level of evidence as a randomised control trial. The authors have designed the study with periods of non-treatment to reduce a wash-out effect of intervention. The results of this trial were reported in two separate papers, one examining the results after the first eight weeks of the trial (after the initial ‘no intervention’ period, one treatment period, and one post ‘intervention no treatment’ period), with another reporting the results after the first twelve weeks of this trial (inclusive of the initial ‘no intervention’ period, two ‘intervention’ periods and the ‘no intervention’ period between the two ‘intervention’ periods). The outcome measures included pain and activity. The outcome of pain was measured with the Borg’s CR-10 scale during two different sporting activities (A = most painful activity, B = less painful activity), and the Engstrom activity index was utilised to measure the participant’s physical activity intensity. The authors reported lower levels of pain with the two self-selected activities for the orthoses compared to the heel raises (activity A odds ratio (95% CI): 0.22 (0.15, 0.34), p < 0.001, activity B: 0.18 (0.12, 0.27), p < .001). This study was classified as providing Level 2 comparative efficacy evidence supporting the use of orthoses compared to heel raise treatments.

The second randomised control trial [40] was a prospective intervention design with a sample size of 45 participants. This trial examined the use of orthoses over a 4 week period compared to a ‘no treatment’ control group with outcomes measured at the beginning and end of the four week ‘intervention’ period. The intervention referred to as a heel cup with a brim, though the picture of these orthoses included in this manuscript also indicated some medial arch support. The construct of pain was captured in this trial through self-reported pain experienced while participating in a chosen ball sport, measured using the Borg CR-10 scale. The authors of this paper reported that there was a significant reduction in pain in sporting activities in the treatment group in this study, however it was not stated which statistical analysis method was used, no confidence intervals or p-values were reported, nor which assessments were being compared in making this statement. Instead, the only data presented was the median values for each group with (for intervention group only) and without (for all participants) orthoses at the pre- and post-intervention period assessments. Use of the orthoses reduced pain with sport at the post intervention assessment to median = 0.5 out of 10 compared to 5 out of 10 for the control group. Physical activity levels were maintained in both groups.

These two randomised trials [38-40] were the highest available evidence reporting the effect of treatment of pain and maintenance of physical activity associated with calcaneal apophysitis. There were a number of areas of uncertainty regarding the methodology of these studies as the study protocol and trial registration status was unable to be obtained. Publishing protocols and registering clinical trials ensures that the research is conducted as required (CONSORT) [48] and that the statistical analysis is transparent [49]. While the cross over design of the first trial incorporated breaks between interventions, given calcaneal apophysitis is a self limiting condition, there is still the possibility that there was a continued wash out effect that carried through the intervention groups. The randomised trials [38-40] as reported, have statistical analysis concerns that limit the validity of the results reported. The completed interim analysis of both Perhamre 2011a and 2011c [38,40], increased the risk of a type 1 error [50]. The final randomised trial [39] also had areas of statistical concern as no information regarding which statistical test used was given and there was no indication of the precision of the estimate provided.

#### Heel lifts

The use of heel lifts was reported in many of the studies [17,38,39,41,44]. All of the studies reported that heel lifts decreased pain, though many of them used these concurrently with other treatment modalities such as stretching and ice [17,41,44] and were unable to provide results regarding the heel raises’ efficacy alone.

The randomised trial previously mentioned [39] examined the use of heel raises within the group results of the crossover randomised trial. The heel raise used within this study was a 5 mm cork wedge covered with a thin elastic surface, and was reported to lift the heel 5 mm in mid stance and push off. The design of this trial permitted participant comparison of pain reported when using no intervention compared to pain reported when using the heel raises. Results for this comparison indicated that pain levels were higher when using no intervention compared to when using the heel raises. Odds ratios (95% CI) for activity A of 2.32 (1.69, 3.19), p-value < .001 and for activity B of 2.29 (1.67, 3.16), p < .001 were reported where use of the heel raise was the reference value. This data was classified as providing Level 2 evidence of efficacy of heel raises for reducing pain with activity over no treatment, however the finding arose from a pre-post within-group comparison, these results should be viewed with caution.

#### Concurrently applied therapies

Many authors also incorporated strategies to both minimise inflammation i.e. icing and active rest, together with the minimisation of the proposed biomechanical contributing factors i.e. gastrocnemius and soleus static stretch as standard, usual care treatments. No studies were identified that examined these modalities as isolated treatment entities. It is not known whether concurrent application of these treatment approaches when investigating other treatment modalities introduces treatment effect-diluting or moderating (interaction) effects.

## Discussion

This review has identified that there has been little methodologically rigorous evidence generated to support treatment options for calcaneal apophysitis. The available evidence indicated orthoses with a brim (heel cup) and medial arch support was more effective in reducing pain in sporting activities (Level 2 evidence) compared to heel raises or no treatment. There was also support for heel raises reducing pain in sporting activities (Level 2 evidence) compared to no treatment. Taping also appeared to have some immediate pain relief benefit (Level 3 evidence). Meta analysis was not undertaken due to the variability of data collected during this review, therefore our conclusions have been based upon a critical, narrative synthesis.

The findings of this review might be able to help shed light over the causative mechanism of calcaneal apophysitis. It is widely accepted that calcaneal apophysitis is a self-limiting condition related to physiological changes at the calcaneal apophysis as children transition to adolescence. Given the causative mechanisms of calcaneal apophysitis are still unknown, it was not surprising to find a variety of treatment approaches advocated within the literature [15]. The treatment options reported within the literature and their success may be based on a number of theories of causative factors. Many researchers and clinicians continue to support the same baseline interventions such as ice and restriction of sports. The effectiveness of additional treatment options such as taping, heel lifts and orthotics may be based on the supposition [51] that calcaneal apophysitis is the result of either an increased tractional pull at the calcaneal apophysis or from increased impact forces at the plantar surface of the heel.

One causative factor of increased tension or shortness of the achilles tendon may be due to the rapid growth in adolescents. This soft tissue change may have the potential to place an interim strain or traction on the apophysis at the insertion [8,42], thus a simple heel raise has been advocated to reduce this strain when footwear is worn [17,38,39,41,44]. Similarly, there may be increased tension or shearing force at the insertion of the achilles tendon due to abnormal biomechanics at the foot and ankle. During gait, the gastrocnemius and soleus muscles provide a plantarflexion mechanism at the ankle while eccentrically contracting at midstance, exerting forces on the subtalar joint [51,52]. If there are abnormal forces going through the foot, the provision of a heel raise may reduce the load in the achilles tendon [53] or an inverted type of orthotic device has the potential to reduce the supination moment [54] and limit pronation; both items reducing the tractional pull at the achilles tendon calcaneal junction. A combination of these theories may also be plausible.

It is not known exactly how calcaneal apophysitis develops and if there is biomechanical abnormalities within the foot that results in active children having heel pain. The resultant success in pain relief an orthotic device may come into play when used on a foot with an abnormal windlass mechanism. The windlass mechanism is the theory that describes how the plantar fascia and achilles tendon interact during gait for propulsion. The plantar fascia (windlass) is like a rope or cable attached to the distal and inferior aspect of the calcaneus and the proximal and inferior aspect of the metatarsophalangeal joints. During the propulsion phase of the gait cycle, the windlass activates and shortens the plantar fascia at the metatarsals, therefore shortening the distance between the calcaneus and metatarsals. This in turn elevates the medial longitudinal arch [55]. It is possible what in a foot with calcaneal apophysitis, the rearfoot valgus position may be impacting the windlass mechanism and changing the force required at the achilles tendon for normal gait. The use of an orthotic has demonstrated ability to influence the rearfoot position [23,41] and therefore may positively impacts the windlass mechanism; this in turn may reduce the loading required at the achilles tendon for normal and pain free gait in the symptomatic child.

Impact forces or increased plantar pressures at the calcaneal area may cause repetitive impact forces during heel strike, further traumatising the apophysis [8,56]. If this is also a contributing cause of the apophysitis, then the use of an orthotic with a heel cup may centre the calcaneal soft tissues (fibro fatty padding), increasing the cushioning in this area and resulting in pain relief [40]. Likewise, a heel raise is often manufactured from material with shock absorbing properties, again providing some form of cushioning at the plantar surface of the heel.

No studies identified in this review examined the use of ‘off the shelf’ or prefabricated orthotics which have also been known to reduce foot pain in adults with plantar fasciitis [57]. There have been no studies investigating the use of prefabricated orthotic devices in neurologically normal children with foot pain and it was surprising these were not utilised within the studies considering the increased availability to health practitioners and their relative low cost compared to custom made orthotics.

It also appears from the literature that children who play competitive sport were more likely to experience calcaneal apophysitis [41,45,58,59]. This is a sub-section of the general population, therefore extrapolation of the treatment approaches and effectiveness demonstrated within these studies to children who participate in normal sporting activities levels may not be appropriate. While orthoses [38-40] were more effective in pain reduction during participant-identified activities compared to heel raises, this may be due to the high impact on the apophysis of many hours of training or an early sporting injury due to abnormal biomechanics and strain. In the child who plays normal school sports or minimal sport after school, the use of a heel lift may be just as effective in pain relief.

Overall, the treatment options reported, both work on similar principles, however the level of effectiveness may actually be based on the original cause of the pain. A regular foot posture and normal foot biomechanics may only require a heel lift to relieve pain, yet the child who has additional foot posture changes may require some type of orthotic with or without a heel lift to improve foot function. Additional domains need to be explored to better understand efficacy of treatment options, including: establishing the level of sport played by children who demonstrate this pain, determination of any ankle equinus and understanding if there is a particular foot type that is more receptive to one treatment over another.

## Conclusion

Calcaneal apophysitis is a condition that may present in children and a cause of primary health care presentations. There has been limited high quality evidence identified to guide treatment approaches. While it appears from the evidence that the use of orthoses and heel lifts give effective pain relief; the studies promoting either treatment modality have many study design concerns. Primary care practitioners should exercise caution when heeding this result. A randomised control trial incorporating short and long-term effects of appropriate treatment modalities is indicated and a study design for this has been published [60]. This research will strengthen the evidence of effectiveness in the reduction of pain and the maintenance of activity in children who present with calcaneal apophysitis.

## Competing interest

The authors declare that they have no competing interests.

## Authors’ contributions

All authors (AMJ, CW, TPH) contributed to the conception and design of this systematic review. AMJ and CMW completed the systematic review and scoring of the selected articles. All authors have contributed to the analysis and have read and approved the final manuscript.
